# On the Preparation of Hydrargyrum Cum Creta, and Hydrargyrum Cum Magnesia of the Dublin Pharmacopœia

**Published:** 1848-11

**Authors:** M. Donovan

**Affiliations:** M. R. I. A.


*On the Preparation of Hydrargyrum cum Creta, and Hydrargyrum cum Magnesia of the Dublin Pharmacopœia.* By M. Donovan, Esq., M. R. I. A.—The hydrargyrum cum creta of the Dublin Pharmacopœia has been known for a century under the name of mercurius alkalisatus. About half a century since it was no longer in use, and was all but forgotten in this country, until the appearance of the Dublin Pharmacopœia of 1807, when it was revived, and a duplicate preparation unnecessarily introduced, in which the only difference was the substitution of magnesia for chalk. A process was given for both of these preparations, which consists in triturating two parts each of mercury and manna with a few drops of water until the metallic particles disappear. An eighth part of chalk or common magnesia is then to be thoroughly mixed in; warm water is to be added in order to wash away the manna; and the ablution is to be repeated several times. Up to this period the process goes on smoothly enough: but we are then directed to mix in seven-eighths more of chalk or magnesia with the wet sediment. Here lies the difficulty: the moment the trituration is resumed, the moisture, which kept the minute metallic globules from coalescing, being absorbed by the magnesia just added, running mercury begins to reappear, and shortly it amounts to the original weight as nearly as could be expected. I once had a trial made of two ounces of mercury which was triturated with manna-syrup for thirty days, during three hours each day, yet at the end of that period, on adding the magnesia, mercury instantly reappeared in globules, and without any difficulty more than an ounce of metallic mercury was recovered. But this was far from being the total obtainable quantity. In another case, sixty grains of metallic mercury were triturated with manna-syrup during two hours every day for twenty days; yet, at the end of that time, no less than fifty-four grains of running mercury were recovered. It must be supposed that there was more metallic mercury present which could not be recovered, owing to the very great ratio of magnesia which entangled the minute globules. In short, not more than three grains of mercury could have been oxidated, if there was any, which I much doubt, for some of the missing quantity must have been the impurities of the mercury, and for other reasons presently to be assigned. But even if there were three grains oxidated, an ounce of mercury would, according to the pharmacopœial process, and at the rate above mentioned, require nine years for its total oxidation.

It is almost inferrible *à priori* that, in this preparation, the mercury is not and could not be oxidated. Each globule is enveloped in a coating of viscid syrup, which adheres with force, and effectually prevents the contact of the atmosphere, even although the globules were at the surface. Whence, then, can it derive its supply of oxygen? the metal is incapable of decomposing water, and therefore cannot be supplied from this source.

The impracticability of preparing the hydrargyrum cum magnesia, or the hydrargyrum cum creta, by the formula of the Dublin Pharmacopœia, has compelled apothecaries to obtain their supply by other means than preparing it themselves. The apothecary purchases from his druggist, and the druggist from the manufacturer, who prepares it according to his own formula.

Being anxious, a few years since, to discover how the manufacturers manage to prepare these articles, I procured samples from several sources and examined them with some care. I found that the samples generally contained about half the quantity of mercury which the Pharmacopœia directs, and that it was in the metallic state.

This and other considerations induced me to try a simple process for the production of these preparations. In the first place, I ascertained that the circuitous process of first extinguishing the mercury by means of manna, and washing the latter away, was not attended with any advantage; that simple trituration of the magnesia or chalk answered the purpose of extinction completely, and with very little trouble, provided that one particular was attended to—namely, a proper ratio of mercury to chalk. If the ratio of the Pharmacopœia be employed, it will be found that the extinction of the mercury, proceeding rapidly at first, will by degrees become very slow, and that a small quantity of running mercury will remain, the extinction of which will cause excessive trouble. This fact at once suggested to me that if the quantity of chalk or magnesia were increased, the trouble of the process would be proportionally diminished. Accordingly, when I triturated equal weights of mercury and chalk, I found that the whole of the mercury disappeared in about three hours trituration:

and this is obviously the process of the manufacturers. Their preparation is not much more than half the specific gravity of what we obtained by the pharmacopœial process, but it is much superior for reasons that will presently appear.

The hydrargyrum cum creta, or cum magnesia, is frequently prescribed in pills, and it is common to direct the mass to be made with mucilage or syrup. But when the ratio of mercury to chalk or magnesia, directed in the Pharmacopœia, has been observed, as soon as mucilage or syrup is added and mixed, almost the whole of the mercury reappears in large globules: and hence either of these simple additions may actually render these medicines powerless. Aqueous liquids will produce the same effects. Conserve of roses, if added in so small a quantity as to form a pilular mass, will do the same injury: but if in quantity sufficient to make the mass very soft, the necessary portion of conserve, being added *at once*, the mercury does not reappear. The most certain medium for forming a mass is very soft extract of gentian, or some other extract which can communicate body. It is hazardous for a prescriber to direct this powder as an ingredient in pills which contain other articles, as it is scarcely possible to foresee in what case it may be rendered utterly inert. The most certain formula is a *soft* bolus, made with conserve of roses previously softened with syrup. If the bolus be made tough, even with these ingredients, the mercury will in all probability be revived.

When hydrargyrum cum creta, or cum magnesia, is prepared with twice as much of the earthy carbonate as the Pharmacopœia directs, the foregoing inconvenience and injury are lessened, but are not obviated. Mercury will generally reappear when the powder is moistened, but its quantity will be much less, and by perseverance in mixing with the other materials, if any, the mercury may be again extinguished. This and the greater facility of production are the grounds of preference to which allusion was just now made, in describing the process of the manufacturer.

I have heard persons declare that they had succeeded in preparing these powders rigidly according to the Pharmacopœia; I have solicited the favour of a sample, but as yet without having obtained it.

Notwithstanding the existence of the mercury in the metallic state in hydrargyrum cum creta, or cum magnesia, even when rightly prepared, we are not to conclude that these preparations are inert; on the contrary, they sometimes evince considerable activity on certain constitutions. The mercury exists in the metallic state even in the best prepared mercurial ointment, yet no one doubts its efficacy. The vulgar opinion that metallic mercury is inert, is erroneous, unless the assertion be confined to running mercury. When minutely divided, it is far from being inactive; witness the ptyalism, often alarming, produced by fumigations, in every one of which the mercury is in the metallic state, no matter whether the fumigating substance had been an oxide placed on a hot iron, or a sulphuret mixed in the substance of a candle.

The complaint against the hydrargyrum cum creta, or cum magnesia, is that the process of the Dublin Pharmacopœia is very troublesome; that it does not succeed; that the resulting powder, when employed in extemporaneous pharmacy, changes its nature more or less, is sometimes very active, sometimes inert, and produces great inconvenience by the revival of a variable quantity of running mercury Besides all this, no two manufacturers employ the same ratio of materials, as will be evidenced by analysing different samples.

The question may be asked, what is the use of making these powders into pills: they are but another form of blue pill, the mercury is exactly in the same state in both; whatever one does, when exhibited as a remedy, so must the other; with only this difference, that one is highly inconvenient both in preparation and exhibition, and the other unexceptionable in either way.

In fine, I suggest that if either of these powders is to be retained in the forthcoming Pharmacopœia, and one of them is quite sufficient, the process should be remodelled. Dry trituration of the materials will suffice, and the ratio of the absorbent earth employed should be doubled, if not tripled.—*Dublin Medical Press.*

